# Significant progressive heterobeltiosis in banana crossbreeding

**DOI:** 10.1186/s12870-020-02667-y

**Published:** 2020-10-27

**Authors:** Michael Batte, Moses Nyine, Brigitte Uwimana, Rony Swennen, Violet Akech, Allan Brown, Helena Persson Hovmalm, Mulatu Geleta, Rodomiro Ortiz

**Affiliations:** 1 International Institute of Tropical Agriculture (IITA), P.O. Box 7878, Kampala, Uganda; 2 Department of Plant Breeding, Swedish University of Agricultural Sciences (SLU), P.O. Box 101, 23053 Alnarp, SE Sweden; 3 Department of Plant Pathology, Kansas State University, Manhattan, KS 66506 USA; 4 International Institute of Tropical Agriculture (IITA), C/o The Nelson Mandela African Institution of Science and Technology (NM-AIST), P.O. Box 447, Arusha, Tanzania; 5 Laboratory of Tropical Crop Improvement, Katholieke Universiteite Leuven (KUL), Willem De Croylaan 42, 3001 Leuven, Belgium; 6 Bioversity International, Willem De Croylaan 42, 3001 Heverlee, Belgium

**Keywords:** Bunch weight, East African highland banana, Genetic distance, Heterobeltiosis, *Musa* spp., NARITA

## Abstract

**Background:**

Heterobeltiosis is the phenomenon when the hybrid’s performance is superior to its best performing parent. Banana (*Musa* spp. AAA) breeding is a tedious, time-consuming process, taking up to two decades to develop a consumer acceptable hybrid. Exploiting heterobeltiosis in banana breeding will help to select breeding material with high complementarity, thus increasing banana breeding efficiency. The aim of this study was therefore to determine and document the level of heterobeltiosis of bunch weight and plant stature in the East African highland bananas, in order to identify potential parents that can be used to produce offspring with desired bunch weight and stature after a few crosses.

**Results:**

This research found significant progressive heterobeltiosis in cross-bred ‘Matooke’ (highland cooking) banana hybrids, also known as NARITAs, when grown together across years with their parents and grandparents in Uganda. Most (all except 4) NARITAs exhibited positive heterobeltiosis for bunch weight, whereas slightly more than half of them had negative heterobeltiosis for stature. The secondary triploid NARITA 17 had the highest heterobeltiosis for bunch weight: 249% versus its ‘Matooke’ grandparent and 136% against its primary tetraploid parent. Broad sense heritability (across three cropping cycles) for yield potential and bunch weight were high (0.84 and 0.76 respectively), while that of plant stature was very low (0.0035). There was a positive significant correlation (*P* < 0.05) between grandparent heterobeltiosis for bunch weight and genetic distance between parents (r = 0.39, *P* = 0.036), bunch weight (r = 0.7, *P* < 0.001), plant stature (r = 0.38, *P* = 0.033) and yield potential (r = 0.59, *P* < 0.001). Grandparent heterobeltiosis for plant stature was significantly, but negatively, correlated to the genetic distance between parents (r = − 0.6, *P* < 0.001).

**Conclusions:**

Such significant heterobeltiosis exhibited for bunch weight is to our knowledge the largest among main food crops. Since bananas are vegetatively propagated, the effect of heterobeltiosis is easily fixed in the hybrids and will not be lost over time after the release and further commercialization of these hybrids.

## Background

Bananas and plantains (*Musa* spp. L.) are important food and cash crops to millions of people in the tropical and subtropical regions of the world [1]. They are grown in more than 135 countries. In India, the largest banana producer, the crop occupies 20% of the area under fruit crops. Bananas and plantains rank among the most important food crops in the developing world [2]. In Uganda, matooke (2*n* = 3*x* = 33 chromosomes) and other bananas are grown by at least 75% of the farmers and cover an estimate of 38% of the total land under crops [3]. However, the production has declined over the past three decades due to mainly declining soil fertility and drought [4], plus pests and diseases. The most economically important pests for bananas in the Great Lakes region of Africa are the burrowing nematode (*Radopholus similis*) and banana weevil (*Cosmopolites sordidus*). The diseases are caused by pathogens which thrive in tropical conditions, the most important of which are *Xanthomonas vasicola* pv. *musacearum* (formerly *Xanthomonas campestris* pv. *musacearum)* leading to banana bacterial wilt [5]*, Pseudocercospora fijiensis,* causing black Sigatoka or black leaf streak disease [6, 7], and *Fusarium oxysporum* f. sp. *cubense* causing fusarium wilt or Panama disease [8]. Breeding of resistant/tolerant cultivars is the most sustainable intervention for banana health management [9–11]. However, plant breeding is a long process requiring efficient selection of suitable parents with desired traits to produce superior hybrids [12].

Utilization of heterosis or heterobeltiosis can speed up the process of generating superior hybrids. Heterosis, or hybrid vigour, is the superiority of the hybrid for a certain trait over the mean of the parents, whereas heterobeltiosis is a form of heterosis where the hybrid is superior to its best performing parent [13]. Jones [14] defined heterosis as the expression of dominance deviation, a variance from mid parent value, which may be explained by the additive effects of several desired dominant alleles, or as “overdominance,” the combined effect of (two) different alleles at the same gene locus, or a combination of both. From the definitions, heterobeltiosis helps a breeder to make more stringent selections than heterosis, as also reported by Lamkey and Edwards [15]. Both positive and negative heterosis can be useful depending on the breeding objectives. Generally, positive heterosis is very useful when selecting for yield and its components, whereas negative heterosis is desired when selecting for short plant height and fast or early cycling [15, 16]. Gowda et al. [12] reported that selection of promising parents to obtain superior hybrids primarily depends on the predominance of the genes for the additive effect due to heterosis and heterobeltiosis.

The underlying genetic and molecular mechanisms of heterosis remain unknown [13]. Some of the theories for heterosis include dominance, over-dominance and epistasis [17, 18]. Tao et al. [19] reported that it is possible to efficiently screen for superior parents and predict the heterosis of parental combinations. They further pointed out that genetic differences between parents are the primary cause of heterosis. Also, the correlation between the genetic distance and heterosis depends on the type of materials. According to Hinze and Lamkey [20], limitations in traditional methods based on geographic origins, genetic relationships, morphological markers and isozymes make the prediction of heterosis difficult. The development of molecular marker techniques is seen as a new and more effective way for heterosis prediction, which will in turn improve the efficiency of hybrid breeding. Van Ginkel and Ortiz [21] reported that heterosis in self-fertilizing crops is often driven by additive and additive × additive gene action. They further argued that this can be relatively easily fixed in homozygous lines, meaning that their seed can simply be re-sown to express the heterosis, unlike non-additive heterosis.

Goff [22] proposed a concept of heterosis which summarizes other theories that were earlier proposed about the physiology of heterosis. It states that “heterosis is a result of allele-specific expression, which favors the expression of the most energy-saving, stable alleles.” In hybrids, alleles at a locus are likely to be different, and there are multiple opportunities for allele-specific expression of the more stable gene product. Hybrids are therefore more efficient in overall energy use than their parents, with most loci in homozygous state and can use the saved energy for other tasks. The saved energy can be invested in higher growth rates compared with the parental lines, a phenomenon we perceive as heterosis. Van Ginkel and Ortiz [21] reported that heterosis due to dominance can be captured in homozygous individuals, as the favorable allele can be present twice in homozygous lines or doubled haploids, unlike heterosis due to overdominance, which involves different alleles of the same gene. More recent research is showing that, in self-fertilizing and some outcrossing crops, dominance is more important than overdominance, implying that additive gene expression exceeds non-additive gene action [23, 24]. However, Goldringer et al. [25] reported a larger epistatic effect than additive genetic variance for grain yield in hexaploid bread wheat (*Triticum aestivum* L.). The more the additive and additive × additive gene actions dominate in hybrids, the more effectively the F_1_ performance predicts the subsequent derived line performance.

Recent research gives an insight in gene actions driving heterosis in various crops. Heterosis for grain yield components appears to be controlled by additive gene action [12], but also, as noted by Beche et al. [13], by additive × additive gene effects. Early research in barley (*Hordeum vulgare* L.) revealed that heterosis in seed yield is due to additive and “homozygous–homozygous” gene effects [26, 27], while heterosis for grain yield in rice (*Oryza sativa* L.) seems to be determined by additive and additive × additive gene action [28–30]. Scanty research results are available about heterosis in bananas [31], though none in the East African highland bananas. The aim of this study was therefore to determine and document the level of heterobeltiosis of bunch weight and plant stature in secondary triploid East African highland banana hybrids, in order to identify potential primary tetraploid hybrids and triploid matooke cultivars to be used as parents of offspring with desired bunch weight and plant stature after crossing diploids with them, thus improving the efficiency of the banana breeding program.

## Results

Broad sense heritability (H^2^) for yield was 0.84, for bunch weight 0.76 and for plant stature 0.0035. NARITA 23 had the highest bunch weight (29.3 kg), followed by NARITA 17 (29.0 kg) and NARITA 18 (28.6 kg) while NARITA 19 had the smallest bunch weight (11.1 kg; Table 1). However, NARITA 17 had the highest yield potential (35.6 t ha ^− 1^ yr ^− 1^), followed by NARITA 23 (35.0 t ha ^− 1^ yr ^− 1^) and NARITA 18 (34.4 t ha ^− 1^ yr ^− 1^) whereas NARITA 19 had the lowest yield potential (14.7 t ha ^− 1^ yr ^− 1^). Similarly, NARITA 17 had the highest heterobeltiosis of 249% versus its ‘Matooke’ grandparent and 136% against its primary tetraploid hybrid parent (Table 1), while NARITA 19 had the lowest heterobeltiosis of 1% against its ‘Matooke’ grandparent and negative (− 34%) against its primary tetraploid parent. NARITA 7 also known as ‘KABANA 6H’, which is the only released NARITA hybrid cultivar in Uganda so far, had a heterobeltiosis of 77 and 15% vis-à-vis its ‘Matooke’ grandparent (female) and primary tetraploid parent. All the 31 NARITAs showed a positive heterobeltiosis for bunch weight, when compared to ‘Matooke’ (3*x* grandparents). Plant stature ranged from 0.16 to 0.21 (Table 2). NARITA 20 had the highest positive grandparent heterobeltiosis for stature (31%), followed by 29285S-20 and NARITA 17 (27%) while NARITA 1 had the highest negative grandparent heterobeltiosis (− 18%). About half of the NARITAs had a negative grandparent heterobeltiosis for plant stature (Table 2).

**Table 1:** Bunch weight (mean ± standard error), genetic distances, yield potential (mean ± standard error), and parent (primary tetraploid hybrid) plus matooke grandparent (triploid) heterobeltiosis for NARITA cultivars

NARITAs	Bunch weight ± SE (kg)	Genetic distance (Parents)	Genetic distance (NARITA: Grandparent)	Yield potential ± SE (t ha ^− 1^ year ^− 1^)	Parent Heterobeltiosis (%)	Grandparent Heterobeltiosis (%)
NARITA 17	29.0 ± 1.5	–	0.4	35.6 ± 2.0	136	249
26666S-1	26.1 ± 1.8	0.9	0.4	28.9 ± 1.2	85	229
NARITA 9	23.8 ± 2.2	0.9	0.6	31.3 ± 3.1	69	201
NARITA 22	23.3 ± 1.9	0.9	0.6	31.8 ± 2.3	65	194
26874S-5	22.6 ± 2.3	–	0.4	27.5 ± 2.8	60	186
26787S-1	22.6 ± 1.7	0.9	–	31.1 ± 2.2	60	185
NARITA 23	29.3 ± 2.5	0.8	0.5	35.0 ± 2.1	173	173
NARITA 14	20.7 ± 2.0	0.9	0.4	27.4 ± 3.1	47	162
NARITA 8	20.3 ± 1.8	0.9	0.5	22.2 ± 1.9	44	157
26337S-11B	27.6 ± 2.6	0.7	0.4	29.2 ± 2.2	63	152
NARITA 4	18.7 ± 1.7	0.9	0.4	23.1 ± 2.1	9	136
NARITA 3	18.7 ± 1.6	0.9	0.3	20.7 ± 1.9	32	136
NARITA 1	18.5 ± 1.7	0.9	0.3	20.1 ± 2.0	31	134
NARITA 2	17.8 ± 1.2	0.9	0.4	24.5 ± 1.9	83	114
NARITA 10	16.7 ± 2.0	0.9	0.6	21.0 ± 2.5	18	111
25974S-19	16.7 ± 1.0	0.9	0.5	24.7 ± 1.1	18	111
NARITA 5	15.6 ± 1.2	0.9	0.3	17.4 ± 1.5	11	97
29792S-14	15.6 ± 2.0	0.8	–	23.0 ± 3.1	11	97
26316S-7	21.4 ± 0.8	0.8	0.4	33.6 ± 1.0	27	96
NARITA 16	15.5 ± 1.7	0.9	0.5	17.6 ± 1.4	10	96
NARITA 13	21.0 ± 1.6	0.8	0.5	30.7 ± 2.5	25	92
NARITA 21	20.2 ± 3.1	0.9	0.4	27.0 ± 3.8	20	84
NARITA 15	14.5 ± 0.9	0.9	0.6	18.2 ± 1.2	−15	84
NARITA 18	28.6 ± 2.0	0.2	0.5	34.4 ± 1.7	129	83
NARITA 7	19.4 ± 2.1	0.7	0.5	19.6 ± 1.7	15	77
NARITA 20	14.3 ± 2.9	0.2	0.5	19.5 ± 4.2	72	72
NARITA 12	18.1 ± 1.1	0.8	0.4	24.2 ± 1.5	7	65
NARITA 11	16.6 ± 0.9	0.8	0.4	23.0 ± 1.6	−1.5	52
29285S-20	16.2 ± 1.7	0.8	0.5	22.9 ± 2.7	−4	48
NARITA 6	18.5 ± 1.2	0.9	0.5	23.2 ± 1.5	124	31
NARITA 19	11.1 ± 1.2	0.5	0.5	14.7 ± 1.5	−34	1

**Table 2:** Plant stature (mean ± standard error), parent and grandparent heterobeltiosis for NARITA cultivars

Genotype	Plant Stature ± SE	Parent Heterobeltiosis (%)	Grandparent Heterobeltiosis (%)
NARITA 20	0.18 ± 0.005	31	31
29285S − 20	0.19 ± 0.004	2	27
NARITA 17	0.18 ± 0.002	3	27
26337S-11B	0.19 ± 0.002	1	26
26316S-7	0.18 ± 0.003	-2	22
NARITA 18	0.21 ± 0.005	16	22
NARITA 13	0.18 ± 0.004	−5	19
NARITA 21	0.17 ± 0.004	−6	17
NARITA 2	0.16 ± 0.002	−1	14
NARITA 7	0.17 ± 0.003	−10	13
NARITA 11	0.17 ± 0.003	−11	12
NARITA 12	0.16 ± 0.004	− 11	11
NARITA 6	0.18 ± 0.004	11	10
NARITA 23	0.19 ± 0.002	6	6
NARITA 19	0.16 ± 0.004	−16	5
26666S-1	0.20 ± 0.011	9	−2
NARITA 9	0.19 ± 0.004	6	−5
NARITA 8	0.19 ± 0.003	1	−9
25974S-19	0.18 ± 0.002	1	−10
NARITA 22	0.18 ± 0.003	0.4	− 10
NARITA 10	0.18 ± 0.003	0.2	−10
29792S-14	0.18 ± 0.004	0.2	−10
NARITA 4	0.18 ± 0.003	−2	−10
26874S-5	0.18 ± 0.004	−0.1	−10
NARITA 15	0.18 ± 0.003	−2	−11
26787S-1	0.18 ± 0.005	−3	−13
NARITA 16	0.18 ± 0.005	−4	−13
NARITA 14	0.18 ± 0.004	−4	−13
NARITA 5	0.17 ± 0.003	−7	−17
NARITA 3	0.17 ± 0.003	−8	−17
NARITA 1	0.17 ± 0.004	−9	−18

**Fig. 1:**
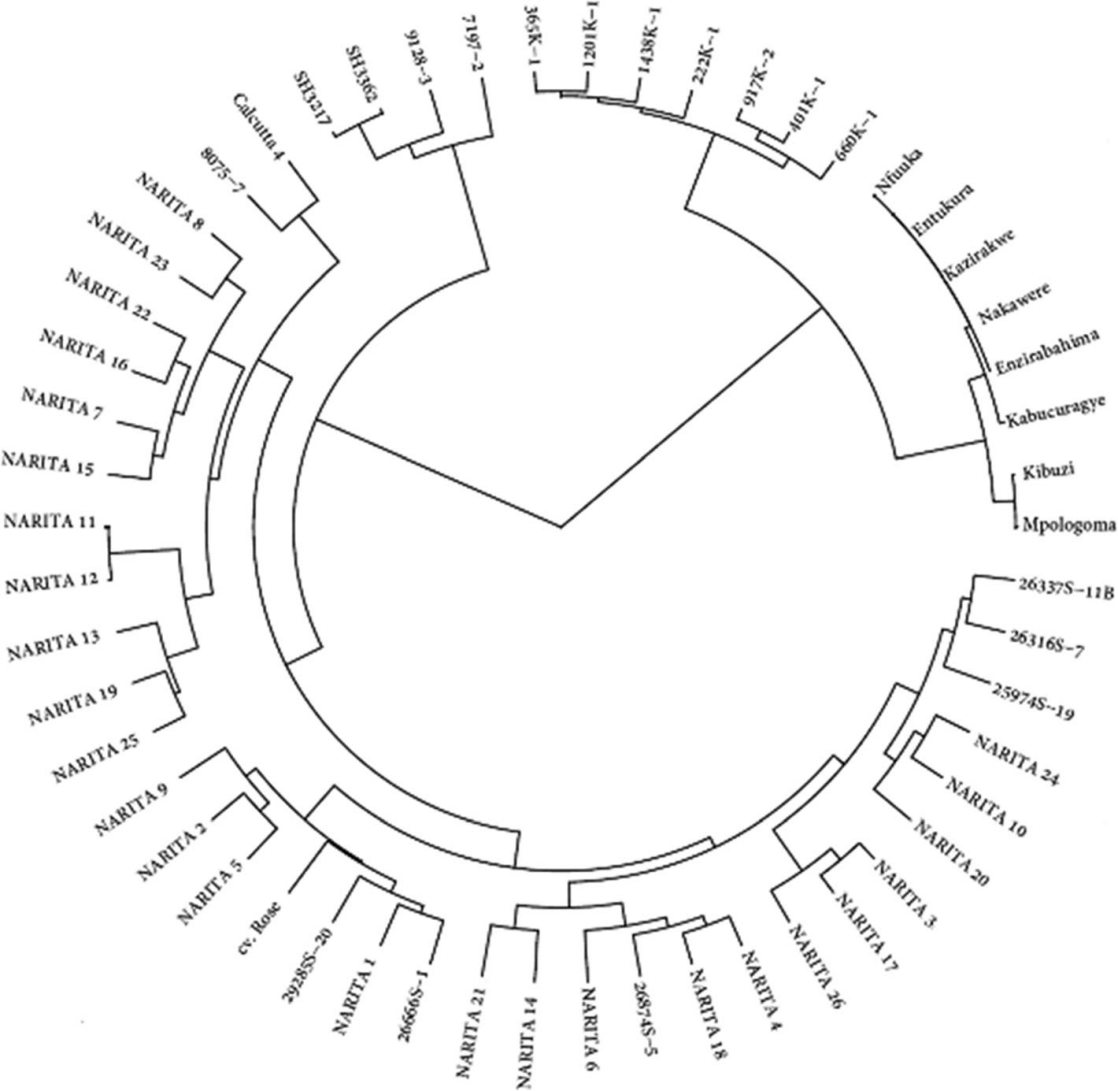
A cladogram showing clustering of NARITAs, their diploid ancestors (parents and 2*x* wild grandparent, ‘Calcutta 4’), primary tetraploid parents, and triploid ‘Matooke’ banana grandparents

**Fig. 2:**
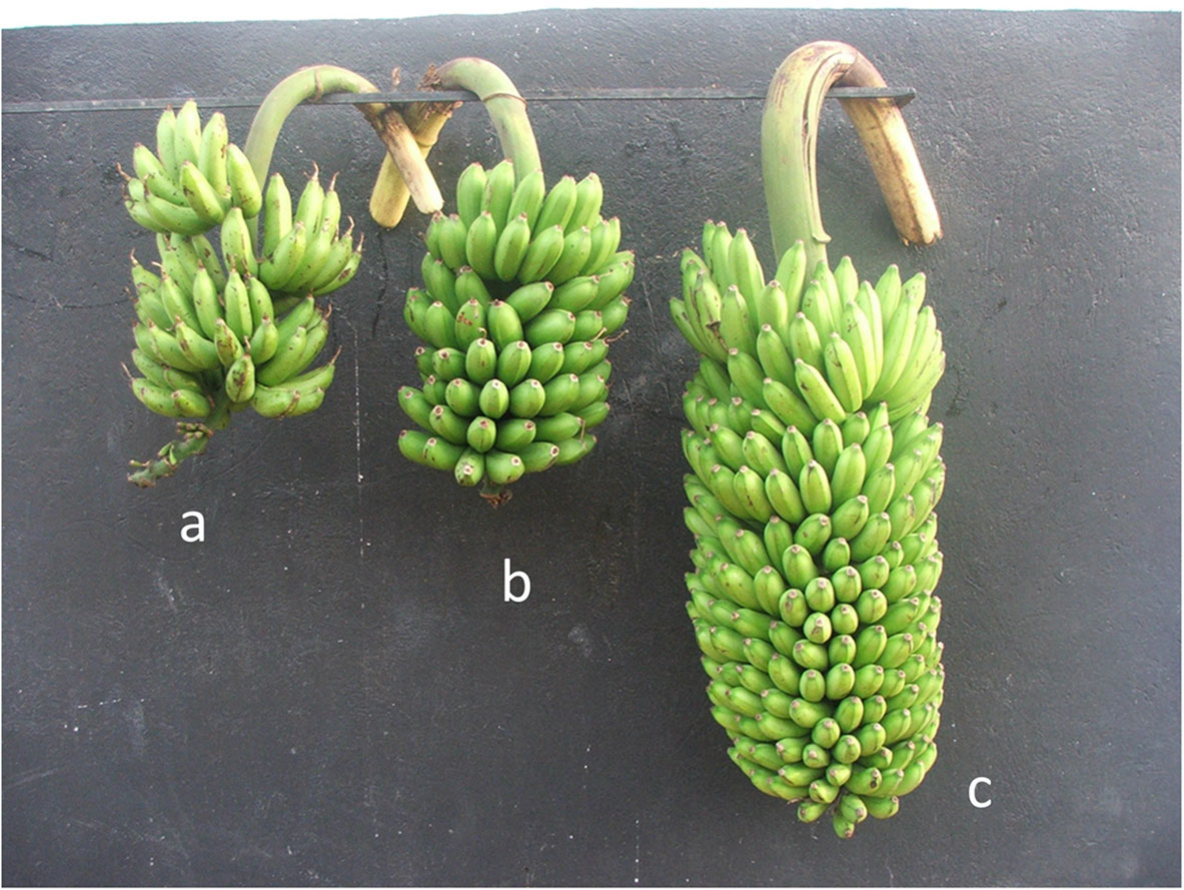
Progressive heterobeltiosis for bunch weight in cross-bred ‘Matooke’ banana hybrids (NARITAs), when grown together with their parents and grandparents in Uganda; **a**: ‘Entukura’ (3*x* female grandparent), **b**: ‘1438 K-1’ (4*x* female parent) and **c**: ‘NARITA 17’ (3*x* hybrid)

There was a positive significant correlation (at 95% confidence level) between grandparent heterobeltiosis for bunch weight and genetic distance between parents (r = 0.39, *P* = 0.036), bunch weight (r = 0.7, *P* < 0.001), plant stature (r = 0.38, *P* = 0.033) and yield (r = 0.59, *P* < 0.001) (Table 3). A significant and negative correlation between grandparent heterobeltiosis for plant stature and the genetic distance between parents (r = − 0.6, *P* < 0.001) was observed (Table 3). In a cladogram (Fig. 1), genotypes of the same known group clustered together such as NARITA cultivars, female parents of NARITAs, male parents of NARITAs and female grandparents of NARITAs, except ‘cv. Rose’ which clustered among the NARITAs between 29285S-20 (a progeny with ‘cv. Rose’ as the male parent) and NARITA 5. There was a significant (*P* ≤ 0.05) progressive heterobeltiosis for bunch weight in bred ‘Matooke’ banana hybrids (NARITA), when grown together across years with their ancestors in Uganda (Fig. 2, Table 1, Table 3). On average, the NARITAs had the highest index of non-spotted leaves (79.3%), followed by their parents (75.8%) and lastly were the grandparents (64.4%).

**Table 3:** Pearson’s correlation coefficients and significance of correlation (*P* ≤ 0.05) between grandparent heterobeltiosis of NARITA hybrids for bunch weight, stature, variance for bunch weight and genetic distances between parents and grandparents, bunch weight, plant stature and yield

	GD parents	GD NARITA: GP	Bunch weight	Plant stature	Yield	HGP. Bunch weight
HGP. Bunch weight	0.39 *P* = 0.036	−0.18 *P* = 0.358	0.70 *P* < 0.001	0.38 *P* = 0.033	0.59 *P* < 0.001	–
HGP. Stature	−0.60 *P* < 0.001	0.12 *P* = 0.539	0.24 *P* = 0.188	0.12 *P* = 0.511	0.33 *P* = 0.068	−0.28 *P* = 0.131
Variance for Bunch weight	−0.10 *P* = 0.5	0.16 *P* = 0.40	- -	- -	- -	- -

HGP=Heterobeltiosis with grandparent (female); GD parents = Genetic distance between NARITA parents; GD NARITA: GP = Genetic distance between NARITA cultivar and grandparent (female)

## Discussion

Genetic factors explained a higher proportion of variance for yield and bunch weight than plant stature. The highest broad sense heritability (H^2^) was recorded for yield (84%) followed by bunch weight (76%), while plant stature had the lowest H^2^ (0.35%). These results differ from those reported by Tenkouano et al. [31] where the heritability estimates of yield components in triploid plantains and derived hybrids were 42% for fruit circumference, 36% for bunch weight and fruit length and zero for number of hands and fruits. However, they argued that this medium heritability enabled yield improvement of individual plants through increased fruit size when recurrent selection was applied. Hence, additional gains could be obtained through crossbreeding, despite the small recombinative heterosis. They further pointed out that diploid males contributed at least twice as much as tetraploid females to the yield of the progeny, implying that paternal phenotype was more predictive of progeny performance for this trait. This finding suggests that great yield gains are likely to be achieved when favorable alleles are accumulated in a diploid male parent. Incorporation of useful genes in the diploids is much easier than in polyploid parents. When these diploid males are crossed with higher ploidy level females, there is a higher probability of recovering hybrid offspring that show heterosis for the desired traits.

All the 31 NARITAs with known pedigrees showed positive grandparent heterobeltiosis for bunch weight and 27 of them also exhibited parent heterobeltiosis. This progressive heterosis, which does not ensue from crossing inbred lines [32], could be a result of favorable allele combinations that are kept in linkage disequilibrium through vegetative propagation in heterozygous parents [33]. The analysis of evolutionary history suggests that bananas underwent instant domestication followed by a few meiosis events; i.e., plant(s) showing high yielding bunch(es) were selected by early farmers, who kept them thereafter by vegetative propagation [33]. Hence, linkats containing favourable gene reassortments, especially in linkage disequilibrium, were preserved through asexual reproduction. This may account for the very large heterosis and grandparent heterobeltiosis noted in the most high-yielding NARITAs, which was above most of other food crops as per available knowledge [34]. It would be interesting to check if further crossbreeding of the NARITAs could maximize progressive heterosis responses resulting in even higher- yielding third generation polyploid hybrids.

Perrier et al. [35] postulated that the rise of cultivated triploid bananas from their direct wild ancestors, *M. acuminata* and *M. balbisiana* among others, was a three-step process. The first step was the anthropogenic circulation of pre-domesticated forms of diploid bananas extracted from the different wild genepools. The second step was the production of edible diploid hybrids, which occasionally produced 2*n* gametes (or showing the sporophytic chromosome number). Finally, sexual recombination among cultivated diploids followed by the fusion of *n* and 2*n* gametes gave rise to the triploid cultivars. The actual number of sexual events that gave rise to the diverse forms of bananas is unknown. However, Bakry and Horry [36] estimated it to be 7 and 14 events while Sardos et al. [37] estimated that the 208 cultivated diploids in their study may have arisen from 117 distinct sexual events, while 80 sexual events were estimated to be at the origin of the 273 triploid accessions based on Diversity Array Technology (DArT) markers. Yet, the East African highland bananas are believed to have arisen from a single ancestral clone that underwent population expansion by vegetative propagation [38].

Plant height is one of the agronomic traits that directly or indirectly influence yield. In cereals like wheat, the increase in yield during the ‘Green Revolution’ was attributed to mutant dwarfing alleles in the *RHt-1* gene which resulted in shorter plants that produced more tillers resulting in an increased number of grains and a reduced lodging by wind and rain [39]. Tall banana plants with slender pseudostems are more prone to wind damage especially after flowering due to the weight of the bunch. Half of the NARITAs expressed negative grandparent heterobeltiosis for plant stature indicating that they were taller and slenderer than the grandparents, which is not desirable for bananas due to high risk of breakage by wind. Lamkey and Edwards [15] and Alam et al. [16] suggested that positive heterosis is desired in the selection for yield and its components, whereas negative heterosis is desired for early cycling and short plant height. In our case however, a positive heterobeltiosis for plant stature was desirable since it indicates that the hybrids are shorter or of the same height as the grandparent but with more robust pseudostems. This is because plant stature in the present study was calculated as a ratio of plant girth at 100 cm from the ground to the total height of the plant at flowering (girth/height). A short plant with a large girth therefore would have a higher value for stature than a tall plant.

Generally, parent heterobeltiosis was lower than grandparent heterobeltiosis for bunch weight for most of the NARITAs. NARITA 17 exhibited the highest parent heterobeltiosis for bunch weight (136%) which was about half of the grandparent heterobeltiosis exhibited by the same genotype. Unlike for the grandparent heterobeltiosis, some NARITAs (NARITA 15, NARITA 11, 29285S-20 and NARITA 19) exhibited negative parent heterobeltiosis for bunch weight implying that on average, the female parents (primary tetraploid hybrids) of these NARITAs had bigger bunches than the NARITAs, whereas the same NARITAs had bigger bunches than their ‘Matooke’ grandparents. Two NARITAs (NARITA 18 and NARITA 6) had their parent heterobeltiosis values higher than grandparent heterobeltiosis for bunch weight. This implies that their parents had smaller bunches than the ‘Matooke’ grandparents. NARITA 23 and NARITA 20 had similar bunch weight for both parent and grandparent heterobeltiosis due to their parentages (Kazirakwe × 7197–2 for NARITA 23 and Entukura × 365 K-1 for NARITA 20), whereby Kazirakwe and Entukura double as grandparents (‘Matooke’) and parents. Nevertheless, progressive heterobeltiosis for bunch weight was demonstrated from ‘Matooke’ grandparents through primary tetraploids to most (27 out of 31) secondary triploid hybrids (NARITAs). Likewise, parent heterobeltiosis was generally lower than grandparent heterobeltiosis for plant stature for most of the NARITAs. NARITA 20 had the highest parent heterobeltiosis for plant stature (31%), a value similar to the grandparent heterobeltiosis. This is due to the parentage of NARITA 20 (Entukura × 365 K-1) whereby Entukura is both a grandparent (‘Matooke’) and parent. NARITA 23 behaved similarly due to the same crossing approach. About half of the NARITAs had negative parent heterobeltiosis for plant stature, a phenomenon similar to that for grandparent heterobeltiosis for the same trait. This implies that the majority of NARITAs were on average taller than their parents and grandparents.

Although there is no unifying theory to explain the phenomenon of heterosis, several mechanisms such as genetic diversity, overdominance, epistasis, and purging of deleterious alleles through heterozygosity have been tested in different models and linked to observed heterosis in complex traits [24]. In the present study, we observed a positive significant correlation between grandparent heterobeltiosis for bunch weight and genetic distance between NARITA parents. These results agree with those of Marcón et al. [40] who also reported a positive relationship between genetic distances among parents and heterosis for forage yield in bahiagrass (*Paspalum notatum*). However, these results contradict with what was observed by Tenkouano et al. [31] in triploid plantains and secondary triploid plantain-derived hybrids. They reported that hybrid performance was negatively but not significantly correlated with the genetic relatedness between the parents. Sant et al. [41] and Joyce et al. [42] also reported negative correlations between genetic distance between parents and hybrid performance in elite Indian chickpea (*Cicer arietinum* L.) cultivars and white clover **(***Trifolium repens* L.), respectively.

The correlation between heterosis and genetic distance between the parents has been widely investigated and, in many cases, a positive relationship has been established although not sufficient on its own to explain heterosis. Xu et al. [43] reported that the genetic distance between parents based on microsatellite data was significantly positively correlated with hybrid yield/yield heterosis in maize, but the coefficient of determination was low and therefore it was not possible to predict the yield heterosis. Genetic distance based on microsatellites was significantly positively correlated with yield heterosis in rice, but not significantly correlated with heterosis for other traits [19]. The correlation coefficient was however too low to be used to predict heterosis. Dias et al. [44] also observed a positive correlation between genetic distances based on random amplified polymorphic DNA markers and heterosis for wet seed weight per plant and wet seed weight per fruit in cacao. They suggested using this as a guide when choosing superior crosses.

Beche et al. [13] reported a positive and significant correlation between heterobeltiosis and grain yield per plant in spring wheat. They suggested using heterobeltiosis for indirect selection of a trait which positively and significantly correlates with the heterobeltiosis. In our study, bunch weight correlated positively and significantly with heterobeltiosis for bunch weight. Hence, this information assists in the indirect selection of parents that are likely to produce superior hybrids. For example, the parents of NARITA 17 (1438 K-1 × 9719–7), 26666S-1(917 K-2 × SH 3362), NARITA 9 (917 K-2 × SH 3217), NARITA 22 (917 K-2 × 9128–3) and 26874S-5 (917 K-2 × 5610S-1), which had the highest heterobeltiosis for bunch weight are likely to produce superior hybrids and therefore might be selected for use in future crosses.

In the current banana breeding program, due the sterility of most banana cultivars, the few cultivars which were screened and found to be fertile are the ones being used in the crosses. The selection of parents was based on ability to produce seed rather than the ability to produce good hybrids (breeding value) [11]. Secondly, the program is not cyclic at the moment in that the same triploid and tetraploid parents which were found to be female fertile are used repeatedly in making crosses. This scenario can be looked at as hybrids derived from the same cross combinations are being evaluated.

Genetic distance between parents was significantly but negatively correlated with grandparent heterobeltiosis for plant stature, while the genetic distance between NARITA cultivar and grandparent (female) was positively but not significantly correlated with the grandparent heterobeltiosis for plant stature. This implies that genetic distance cannot be used to predict the stature of the banana plants considered in this study. Bunch weight as well as yield were positively but not significantly correlated with grandparent heterobeltiosis for plant stature. This implies that the plant stature does not affect the bunch size and yield of banana. It is only desirable for the plant to be short to avoid damage by strong wind and also for the pseudo-stem to be strong to support the bunch until maturity. Grandparent heterobeltiosis for bunch weight was negatively and not significantly correlated with grandparent heterobeltiosis for plant stature. This implies that, the cultivar with the highest heterobeltiosis for bunch weight will not necessarily have the highest heterobeltiosis for plant stature. Hence these two traits are not correlated.

As indicated by Xu et al. [42], microsatellite markers showing high polymorphism can be used to assess genetic relationships and are widely used in assessing genetic diversity, identifying germplasm and characterizing population structures. The clustering of accessions in the cladogram based on microsatellite markers (Fig. 1) agreed with the known pedigree information as well as the defined *Musa* groups according to taxonomy. The high genetic variation among the NARITAs was attributed to diverse alleles from the diploid male parents because the 3*x* grandparents and the tetraploid parents clustered together indicating a low genetic diversity among these accessions. Boeven et al. [45] indicated that parents need to be genetically diverse to ensure heterosis in their hybrid offspring. However, genomic-led analysis revealed that diversity does not lead to heterosis [46, 47]. Indeed, there are various reports indicating positive or negative significant correlations between heterosis in hybrid offspring and the genetic distances among their parents. Hence, this association between parental divergence and heterosis does not have to be relevant when pursuing hybrid breeding. Correlations between parental genetic distances and phenotypic hybrid performance have been reported to be very low in most circumstances, which shows that genetic diversity alone is not enough to obtain heterosis. Although the genetic distance does not affect heterosis in a linear fashion, it is still important for obtaining heterosis in crosses. In many circumstances, the expression of heterosis is partly due to genetic diversity which is part of the genomic core for complex interactions of biological pathways that result into increased hybrid vigor.

From our study, it was observed that the NARITAs had the highest mean index of non-spotted leaves (79.3%), which is a measure of the available photosynthetic area, followed by their parents (75.8%) and lastly the grandparents (64.4%). From the above observation, the available photosynthetic area at the onset of fruit fulling is likely to have contributed to improved bunch weight in the NARITA hybrids, which was also noted in primary tetraploid plantain-banana hybrids [48, 49].

## Conclusion

Heterobeltiosis in high yielding banana hybrids was kept after two crossing generations, thus suggesting a progressive heterobeltiosis. Such a significant heterobeltiosis appears to be the largest among the main food crops as per available literature. Since bananas are vegetatively propagated, the effect of heterobeltiosis is easily fixed in the hybrids and will not be lost over time after release and further commercialization of the hybrids. The factors behind heterobeltiosis in banana are yet to be defined. Nonetheless, leveraging on this high heterobeltiosis there is a huge potential to improve banana production by developing high yielding banana hybrids in relatively few crossbreeding cycles.

## Methods

A field experiment was set up in 2015 at Namulonge- Sendusu in Uganda (00^o^31’ 47″ N and 32^o^36′ 9″ E), comprising 34 NARITA cultivars (26 officially named and 8 not yet officially named), their parents, grandparents and local ‘Matooke’ banana cultivars as controls (Table 4). The NARITA cultivars used in this study represent the best hybrids selected in first 20 years of banana breeding by the International Institute of Tropical Agriculture in collaboration with the National Agricultural Research Organization of Uganda. These hybrids were selected due to their bunch size and host plant resistance to black sigatoka being superior to the landraces (‘Matooke’ grandparents). These cultivars were planted following a 7 × 8 rectangular lattice design using two replications, with a spacing of 3 m between rows and 2 m between plants within a row, thereby having a plant density of 1667 plants ha^− 1^. Data for bunch weight (kg) were collected at harvest for three crop cycles. Yield potential (t ha ^− 1^ yr ^− 1^) was calculated as:

$$ \mathrm{YLD}=\mathrm{BW}\times 365\times 1667/\left(\mathrm{DH}\times 1000\right) $$

where YLD is yield potential (t ha ^− 1^ yr ^− 1^), BW is bunch weight (kg) and DH is days to harvest.

**Table 4:** Parentage of triploid (2*n* = 3*x*) NARITAs along with ploidy of parents and grandparents

NARITA	Female parent	Ploidy	Male parent	Ploidy	Grandparent	Ploidy
NARITA 17	1438 K-1	4*x*	9719–7	2*x*	Entukura	3*x*
26666S-1	917 K-2	4*x*	SH 3362	2*x*	Enzirabahima	3*x*
NARITA 9	917 K-2	4*x*	SH 3217	2*x*	Enzirabahima	3*x*
NARITA 22	917 K-2	4x	9128–3	2*x*	Enzirabahima	3*x*
26874S-5	917 K-2	4*x*	5610S-1	2*x*	Enzirabahima	3*x*
26787S-1	917 K-2	4*x*	9128–3	2*x*	Enzirabahima	3*x*
NARITA 23	Kazirakwe	3*x*	7197–2	2*x*	Kazirakwe	3*x*
NARITA 14	917 K-2	4*x*	7197–2	2*x*	Enzirabahima	3*x*
NARITA 8	917 K-2	4*x*	SH 3217	2*x*	Enzirabahima	3*x*
26337S-11B	1201 K-1	4*x*	SH 3217	2*x*	Nakawere	3x
NARITA 4	660 K-1	4*x*	9128–3	2*x*	Enzirabahima	3*x*
NARITA 3	917 K-2	4*x*	SH 3362	2*x*	Enzirabahima	3*x*
NARITA 1	917 K-2	4*x*	9128–3	2*x*	Enzirabahima	3*x*
NARITA 2	401 K-1	4*x*	9128–3	2*x*	Entukura	3*x*
NARITA 10	917 K-2	4*x*	SH 3217	2*x*	Enzirabahima	3*x*
25974S-19	917 K-2	4*x*	SH 3362	2*x*	Enzirabahima	3*x*
NARITA 5	917 K-2	4*x*	SH 3217	2*x*	Enzirabahima	3*x*
29792S-14	917 K-2	4*x*	cv. Rose	2*x*	Enzirabahima	3*x*
26316S-7	1201 K-1	4*x*	SH 3362	2*x*	Nakawere	3*x*
NARITA 16	917 K-2	4*x*	SH 3362	2*x*	Enzirabahima	3*x*
NARITA 13	1201 K-1	4*x*	SH 3362	2*x*	Nakawere	3*x*
NARITA 21	1201 K-1	4*x*	7197–2	2*x*	Nakawere	3*x*
NARITA 15	660 K-1	4*x*	9128–3	2*x*	Enzirabahima	3*x*
NARITA 18	365 K-1	4*x*	660 K-1	4*x*	Kabucuragye	3*x*
NARITA 7	1201 K-1	4*x*	SH 3217	2*x*	Nakawere	3*x*
NARITA 20	Entukura	3*x*	365 K-1	4*x*	Entukura	3*x*
NARITA 12	1201 K-1	4*x*	9128–3	2*x*	Nakawere	3*x*
NARITA 11	1201 K-1	4*x*	9128–3	2*x*	Nakawere	3*x*
29285S-20	1201 K-1	4*x*	cv. Rose	2*x*	Nakawere	3*x*
NARITA 6	222 K-1	4*x*	9128–3	2*x*	Nfuuka	3*x*
NARITA 19	1201 K-1	4*x*	8075–7	2*x*	Nakawere	3*x*
NARITA 24	Unknown	N/A	Unknown	N/A	Unknown	N/A
NARITA 25	Unknown	N/A	Unknown	N/A	Unknown	N/A
NARITA 26	Unknown	N/A	Unknown	N/A	Unknown	N/A

The mean bunch weights and standard errors were calculated and used to determine heterobeltiosis using the formula:

$$ \mathrm{Heterobeltiosis}\ \left(\%\right)=\left[\left(``\mathrm{NARITA}"\mathrm{mean}\ \mathrm{bunch}\ \mathrm{weight}-``3x\kern0.5em \mathrm{Grandparent}"\mathrm{mean}\ \mathrm{bunch}\ \mathrm{weight}\right)/``3x\kern0.5em \mathrm{Grandparent}"\mathrm{mean}\ \mathrm{bunch}\ \mathrm{weight}\right]\times 100 $$

Plant height and plant girth at 100 cm above the ground were measured at flowering. These data were used to estimate plant stature as the ratio of plant girth to height at flowering, which can be interpreted as a measure of the robustness of the pseudo-stem. The mean plant stature and standard errors were calculated and used to determine heterobeltiosis using the formula:

$$ \mathrm{Heterobeltiosis}\left(\%\right)=\left[\left(``\mathrm{NARITA}"\mathrm{mean}\ \mathrm{plant}\ \mathrm{stature}-``3x\kern0.5em \mathrm{Grandparent}"\mathrm{mean}\ \mathrm{plant}\ \mathrm{stature}\right)/``3x\kern0.5em \mathrm{Grandparent}"\mathrm{mean}\ \mathrm{Plant}\ \mathrm{stature}\right]\times 100 $$

Means of 3*x* grandparents were used to calculate heterobeltiosis of hybrids instead of their parents as the parents are not suitable for consumption and therefore not ideal for comparison. Hence, the type of heterobeltiosis calculated above was grandparent heterobeltiosis.

Also, parent heterobeltiosis for bunch weight and plant stature were calculated as above, replacing grandparent with parents’ mean values for bunch weight and plant stature. The grandparent and parent heterobeltiosis for the two traits were compared.

Variance components were estimated using the mixed linear model with restricted maximum likelihood (REML) method as follows:

$$ \mathrm{Model}=\mathrm{lmer}\left(\mathrm{Trait}\sim \mathrm{Block}+\mathrm{Rep}+\left(1|\mathrm{Cycle}\right)+\left(1|\mathrm{Genotype}\right)+\left(1|\mathrm{Genotype}:\mathrm{Cycle}\right),\mathrm{data}=\mathrm{data}\right) $$

Broad sense heritability (H^2^) for yield, bunch weight, and plant stature was estimated using the formula:

$$ \mathrm{Heritability}=\operatorname{var}\ \left(\mathrm{Genotype}\right)/\left[\operatorname{var}\ \left(\mathrm{Genotype}\right)+\operatorname{var}\left(\mathrm{cycle}\right)/\mathrm{no}.\mathrm{of}\ \mathrm{cycles}+\operatorname{var}\ \left(\mathrm{Genotype}:\mathrm{Cycle}\right)/\mathrm{no}.\mathrm{of}\ \mathrm{years}\ \mathrm{for}\ \mathrm{experiment}+\operatorname{var}\ \left(\mathrm{Residual}\right)/\left(\mathrm{no}.\mathrm{of}\ \mathrm{cycles}\times \mathrm{no}.\mathrm{of}\ \mathrm{years}\ \mathrm{of}\ \mathrm{experiment}\right)\right] $$

where var. (Genotype) is the variance component of the genotype, var. (cycle) is the variance component of the cycle, var. (Genotype: Cycle) is the variance component of the interaction between genotype and cycle, and var. (Residual) is the variance component of the residual. Since the data were recorded for 3 cycles during a period of 3 years, the formula used was:

$$ \mathrm{Heritability}=\operatorname{var}\left(\mathrm{Genotype}\right)/\left[\operatorname{var}\left(\mathrm{Genotype}\right)+\operatorname{var}\left(\mathrm{cycle}\right)/3+\operatorname{var}\left(\mathrm{Genotype}:\mathrm{Cycle}\right)/3+\operatorname{var}\left(\mathrm{Residual}\right)/9\right] $$

The number of functional leaves at flowering and the youngest leaf spotted at flowering were measured and used to compute the index of non-spotted leaves, which is a measure of the photosynthetic area available at the start of fruit filling. The index of non-spotted leaves was calculated using the formula:

$$ \mathrm{INSL}=\left(\mathrm{YLS}-1\right)/\mathrm{NFL}\times 100 $$

Where INSL is the index of non-spotted leaves, YLS is the youngest leaf spotted due to black Sigatoka and NFL is the number of functional leaves.

### Genotyping using SSR

To determine the effect of genetic distance on heterobeltiosis in banana, we genotyped the advanced hybrids (NARITAs), their parents and grandparents using simple sequence repeat (SSR) markers or microsatellites. Fresh young cigar leaf samples were collected from the field in Uganda and shipped under the cold chain to the Institute of Experimental Botany, Olomouc, Czech Republic. Leaf samples were lyophilized in Falcon tubes and stored at room temperature. Approximately 20 mg of lyophilized tissue was crushed into powder in 2 ml Eppendorf tubes using a tissuelyzer. DNA was extracted from tissue powder using NucleoSpin Plant II kit (Macherey-Nagel, Germany) following the manufacturer’s instructions. The concentration and quality of DNA was assessed by a NanoDrop ND-1000 spectrophotometer. The working concentration of DNA was adjusted to ~ 10 ng/μl. Genotyping was done using 19 informative *Musa* SSR primers following the protocol of Christelová et al. [50]. Two independent rounds of PCR were performed followed by fragment analysis. Alleles for each sample were inspected in a GeneMarker v1.75 (Softgenetics, State College, PA, USA) and manually scored for presence (1) or absence (0) only when concordance of alleles between PCR runs was observed. In case a sample showed inconsistency in allele sizes between two PCR runs, a third PCR run was performed to confirm the alleles. Squared Euclidean distances between genotypes were calculated using the dist function of R software v3.4 [51]. The Euclidean distances were scaled to vary between 0 and 1 by dividing with the maximum distance. Hierarchical clustering based on the ward. D2 method [52, 53] was done with the function hclust provided in R package ‘ape’. Pearson’s correlation coefficients between grandparent heterobeltiosis for bunch weight and the genetic distances between parents of NARITAs, genetic distance between NARITAs and their grandparents (female), yield, bunch weight and plant stature were calculated. Also, Pearson’s correlation coefficients between grandparent heterobeltiosis for plant stature and the genetic distances between parents of NARITAs, genetic distance between NARITAs and their grandparents (female), yield, bunch weight, plant stature and grandparent heterobeltiosis for bunch weight were calculated using R software v3.4 [51]. A correlation between the NARITA parents’ genetic distance and variance for bunch weight and the correlation between the genetic distance between NARITA cultivar and grandparent and variance for bunch weight were as well calculated.

## Data Availability

The datasets generated and analyzed during the current study are available from the corresponding author on reasonable request.

## Abbreviations

BW: Bunch weight
DH: Days to harvest
GD: Genetic distance
GP: Grandparent (female) of NARITA
HGP: Heterobeltiosis with female grandparent
INSL: Index of non-spotted leaves
NFL: Number of functional leaves
PCR: Polymerase chain reaction
SE: Standard error
SSR: Simple sequence repeat
Var: Variance
YLD: Yield potential
YLS: Youngest leaf spotted
